# ﻿*Glochidionyangchunense* (Phyllanthaceae), a new species with discoid flowers from Guangdong Province, China

**DOI:** 10.3897/phytokeys.239.118411

**Published:** 2024-03-21

**Authors:** Zhu-Qiu Song, Gang Yao

**Affiliations:** 1 Key Laboratory of Plant Resources Conservation and Sustainable Utilization, South China Botanical Garden, Chinese Academy of Sciences, Guangzhou, China South China Botanical Garden, Chinese Academy of Sciences Guangzhou China; 2 South China National Botanical Garden, Guangzhou, China South China National Botanical Garden Guangzhou China; 3 College of Forestry and Landscape Architecture, South China Agricultural University, Guangzhou, China South China Agricultural University Guangzhou China

**Keywords:** Karst, Malpighiales, Phyllantheae, *Phyllanthodendron*, Taxonomy

## Abstract

*Phyllanthodendron* can be readily morphologically distinguished from *Glochidion*, but recent molecular evidence showed that *Phyllanthodendron* is paraphyletic due to *Glochidion* being nested within it. In this study, a new species of the former *Phyllanthodendron* is described and illustrated as *Glochidionyangchunense* Z.Q. Song & Gang Yao from the limestone areas of South China. This is a peculiar new species and morphologically distinguished by its discoid flowers, T-shaped disc segments, and glabrous flowering branches. A key to *Glochidionyangchunense* and related species in China is provided here.

## ﻿Introduction

*Phyllanthodendron* Hemsl. was previously considered as a distinct genus (Croizat 1942; Li 1987, 1994; Li and Gilbert 2008; Xia and Tong 2018), or treated as a section or a subgenus of the genus *Phyllanthus* L. (Beille 1927; Chantaranothai 2007; Webster and Carpenter 2008; Bouman et al. 2018). However, several molecular studies revealed a highly supported sister relationship between *Phyllanthodendron* and the genus *Glochidion* J.R. Forst. & G. Forst. (Kathriarachchi et al. 2006; Pruesapan et al. 2012; van Welzen et al. 2015; Luo et al. 2017; Pornpongrungrueng et al. 2017). A more comprehensive molecular phylogenetic study has recently shown that *Phyllanthodendron* is a paraphyletic group with *Glochidion* nested within (Bouman et al. 2021). In a result, *Phyllanthodendron* was formally united with *Glochidion* (Bouman et al. 2022). Currently, *Glochidion* is the largest genus of the family Phyllanthaceae and comprises 300–350 species of shrubs or trees distributed in Asia and Pacific (Yao et al. 2020; Bouman et al. 2022). In the Plants of the World Online (https://powo.science.kew.org/; POWO 2023), 328 accepted specific names are listed under the genus. Within *Glochidion*, three subgenera were elected, i.e. subg. Glochidion, subg. Phyllanthodendron (Hemsl.) R.W. Bouman and subg. Pseudoactephila (Croizat) R.W. Bouman. The two latter subgenera correspond to the previous *Phyllanthodendron* and consist of 19 Asian species, which can be readily distinguished from the subgenus Glochidion by the presence of a floral disc and apiculate anthers (Bouman et al. 2022).

**Figure 1.**
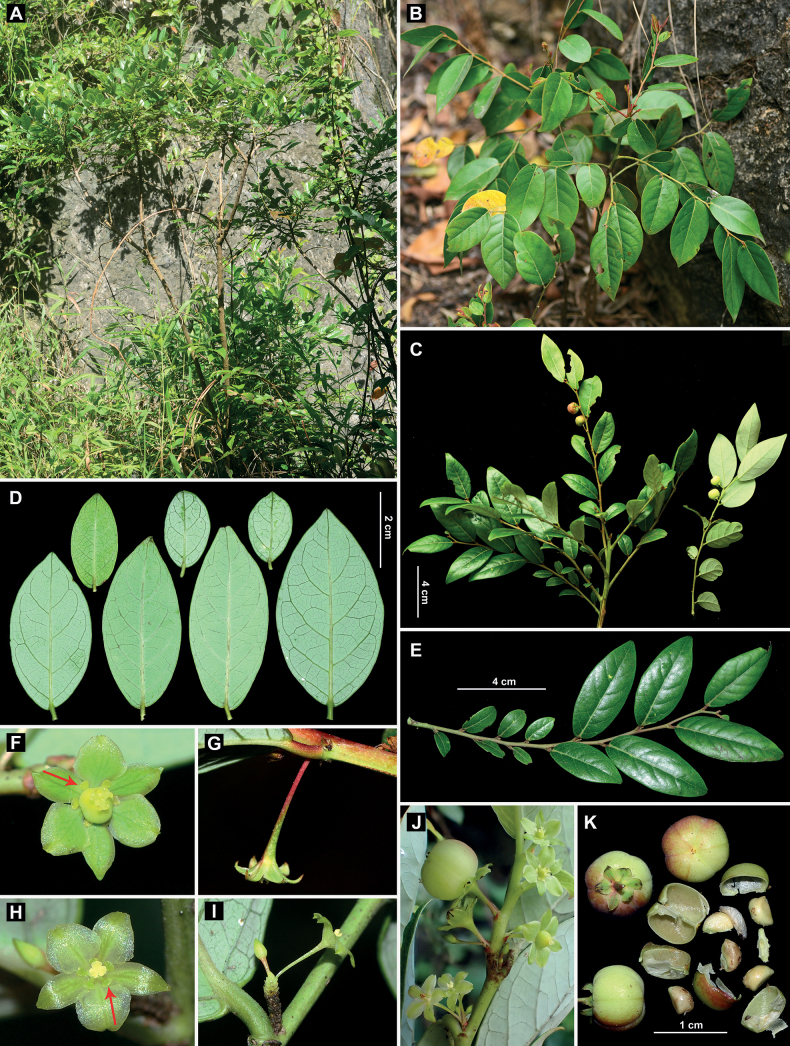
*Glochidionyangchunense* Z.Q. Song & Gang Yao **A** habit **B** detail of the apex of branches **C** fruiting branches **D, E** leaves, adaxial and abaxial surfaces, shapes and sizes **F, G** pistillate flower, front and lateral view **H, I** staminate flower, front and lateral view **J** branches with staminate and pistillate flowers as well as fruit **K** fruits and seeds. Arrows indicate disc segments.

During our field plant investigations in Yangchun City, Guangdong Province, South China, we found an interesting monoecious shrub in a limestone hill. The plant has five (male flowers) or six (female flowers) sepals, three stamens, connate filaments, apiculate anther connectives, 3-locular ovaries, unlobed floral disc segments, and inflated capsules (Fig. 1). These characters are consistent with those of the previous *Phyllanthodendron* and the plant may be a member of Glochidionsubg.Pseudoactephila for lacking the specialized floriferous branchlets, as circumscribed in Bouman et al. (2022). After being compared with all known related species, this plant is proposed as new to science. A full description, color photographs, phenology, conservation status, morphological comparison, and a distribution map of the species are provided here.

## ﻿Material and methods

All specimens of the previous *Phyllanthodendron* (now as Glochidionsubg.Phyllanthodendron and G.subg.Pseudoactephila) kept in the Herbaria CANT, GXMG, GXMI, HITBC, IBK, IBSC, KUN, PE and SYS have been carefully examined by visiting these herbaria, and the images of *Phyllanthodendron* specimens deposited in the Herbaria A, BM, E, K, M, MO, P, US, WU and SZG were also studied. Acronyms for the herbaria follow the Index Herbariorum (Thiers 2023). We also observed the living status of relevant taxa through field investigations and accessing some websites such as Plant Photo Bank of China (https://ppbc.iplant.cn/), Chinese Field Herbarium (https://www.cfh.ac.cn/), and Chinese Union of Botanical Gardens (https://image.cubg.cn/). The distribution map was made by the software ArcGIS 10.2.

## ﻿Result

### ﻿Taxonomic treatment

#### ﻿ Glochidion yangchunense

Taxon classificationPlantaeMalpighialesPhyllanthaceae

Z.Q. Song & Gang Yao sp. nov.

71F013F5-2D4C-5C90-A1BB-B1E32F0F7173

urn:lsid:ipni.org:names:77338772-1

Figs 1 , 2 阳春珠子木

##### Type.

**Figure 2.**
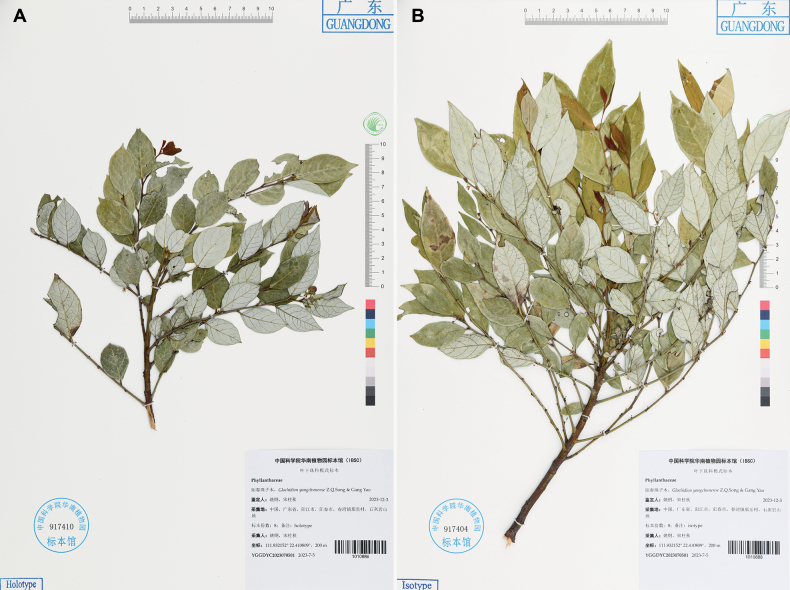
*Glochidionyangchunense* Z.Q. Song & Gang Yao **A** holotype (IBSC1010886) **B** isotype (IBSC1010888). Used with permission.

China. Guangdong Province, Yangchun City, Chunwan Town, Nali village, in limestone hills, 22.410809°N, 111.932152°E, alt. 200 m., 5 July 2023, *Gang Yao & Zhu-Qiu Song YGGDYC2023070501* (holotype: IBSC [IBSC1010886], Fig. 2A; isotypes: IBSC [IBSC1010887, IBSC1010888, IBSC1010889, IBSC1010890, IBSC1010891, IBSC1010892, IBSC1010893]).

##### Diagnosis.

**Figure 3.**
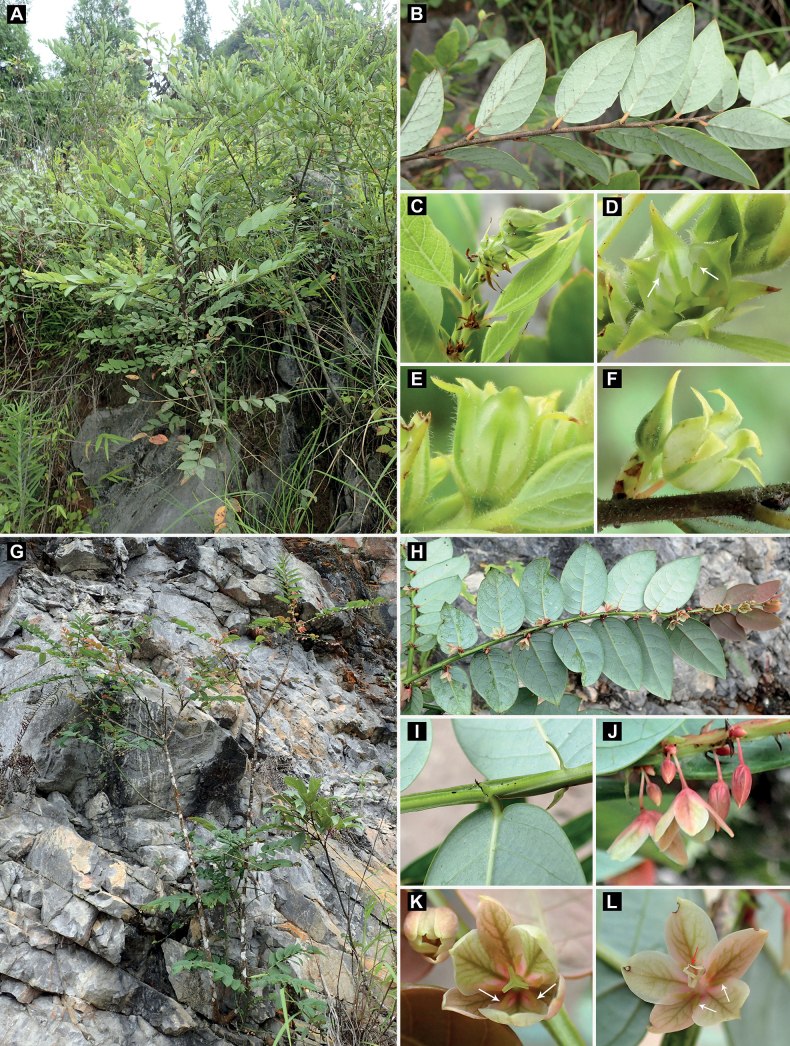
Related species of *Glochidionyangchunense* Z.Q. Song & Gang Yao **A–F***Glochidionanthopotamicum* (Hand.-Mazz.) R.W. Bouman **G–L***Glochidiondunnianus* (H. Lév.) R.W. Bouman **A, G** habit **B, H, I** branches **C–F, J–L** flowers, front and lateral view **H** part of winged branch with stipules **D, K** pistillate flowers **E, F, L** staminate flowers. White arrows indicate disc segments and red arrow indicates stamen.

*Glochidionyangchunense* resembles *Glochidionanthopotamicum* (Hand.-Mazz) R.W. Bouman in general morphology, but much differs from the latter by its glabrous flowering branches (Fig. 1G, I) (*vs.* pubescent flowering branches; Fig. 3C–F), sepals with lateral veins (Fig. 1F, H) (*vs.* sepals without lateral veins; Fig. 3D–F), sepals jointly formed a discoid shape (Fig. 1F–I) (*vs.* sepals jointly formed a urceolate shape; Fig. 3D–F), T-shaped disc segments (Fig. 1F, H) (*vs.* linear disc segments; Fig. 3D), and short stipules (ca. 1 mm *vs.* 3 mm long).

##### Description.

***Shrubs***, 0.5–2.5 m tall, erect, monoecious; stem gray-brown; branches glabrous and terete, but sparsely gray puberulent and slightly angular when young. ***Stipules*** ovate-triangular, ca. 0.8 × 0.6 mm, usually caducous. ***Petiole*** 2.5–4 mm long, sparsely gray puberulent when young. ***Leaves*** simple, alternate, distichous; leaf blades papery to leathery, broad elliptic, elliptic, ovate, or narrowly ovate, length/width ratio 1.5–2.1, glabrous but puberulent on vines below when young; leaf blades at upper part of branches usually larger, 3.5–5.2 × 1.5–3 cm, lateral veins in 6–8 pairs; leaf blades at lower part of branches usually smaller, ca. 1.8–2.5 × 1.2 cm, lateral veins in 3–5 pairs; leaf blades margin entire, slightly revolute, apex acute, rarely acuminate, base sub-rounded; midrib and lateral veins flattened above, slightly elevated below, anastomosing before margins. ***Inflorescences*** axillary, 2–4-flowered; male flowers usually inserted at the lower part of branches, female flowers inserted at the upper part; flowers sometimes crowded in long-pedicelled clustered fascicles; pedicels 6–8.5 mm long, enlarged at apex. ***Staminate flower***: sepals 5 or rarely 6, imbricate, forming a discoid shape, green yellow, midrib elevated on abaxial surface, ovate, 3.3–4.3 × 1.4–2.3 mm, outer sepals lanceolate, inner ones ovate, glabrous; petals absent; disc segments 5, rarely 6, free, T-shaped and expand at apex, slightly greenish yellow; stamens 3, or rarely 4; filaments connate into a terete column, anthers erect, dehiscing longitudinally, connectives usually apiculate. ***Pistillate flower***: sepals 6, imbricate, forming a discoid shape, glabrous, size as in staminate flower; petals absent; disc segments 6, free, T-shaped and expand at apex, slightly greenish yellow; ovary 3-locular; styles 3; stigmas evidently bifid. ***Capsules*** subglobose, 8–10 mm in diam., smooth outside, brownish when mature, fruiting pedicels 6–9 mm long, enlarged at apex. ***Seeds*** obscurely 3-angled or laterally compressed, ca. 4.2–4.5 × 2.7 mm, brownish when mature.

##### Phenology.

Flowering and fruiting from March to December.

##### Distribution and habitat.

**Figure 4.**
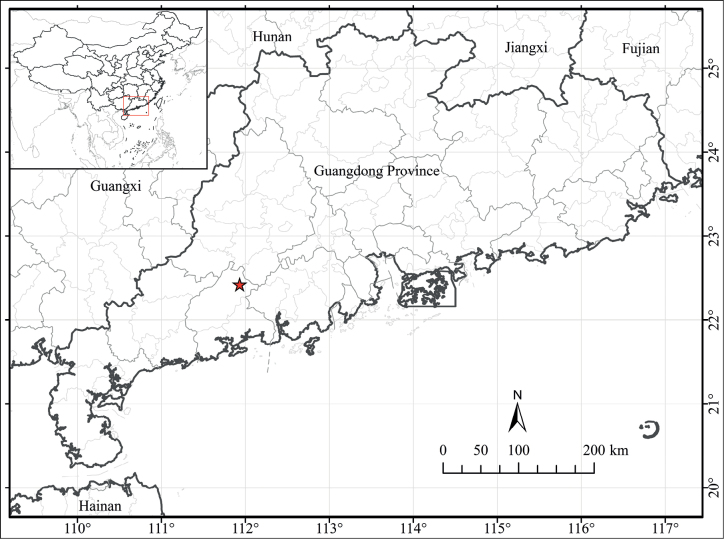
Distribution map of *Glochidionyangchunense* Z.Q. Song & Gang Yao in China (red star).

*Glochidionyangchunense* is currently known only from the type locality, Chunwan Town, Yangchun City, Guangdong Province, China (Fig. 4), and it grows in limestone hills usually at elevations over 200 m.

##### Etymology.

The specific epithet refers to the type locality, Yangchun City in Guangdong Province, China, a hotspot for biodiversity research in Guangdong Province, where multiple new taxa of plant (e.g. *Ilexyangchunensis* C.J. Tseng, Chiritopsissubulatavar.yangchunensis W.T. Wang, *Hedyotisyangchunensis* W.C. Ko & Zhang, *Iteayangchunensis* S.Y. Jin, *Cleyerayangchunensis* L.K. Ling, Alpiniastachyoidesvar.yangchunensis Z.L. Zhao & L.S. Xu, *Symplocosyangchunensis* H.G. Ye & F.W. Xing, *Lithocarpusyangchunensis* H.G. Ye & F.G. Wang, *Crotonyangchunensis* H.G. Ye & N.H. Xia, *Mitreolayangchunensis* Q.X. Ma, H.G. Ye & F.W. Xing, *Heliciayangchunensis* H.S. Kiu, *Primulinayangchunensis* Y.L. Zheng & Y.F. Deng, and *Spiradiclisyangchunensis* R.J. Wang) have been described (see Song et al. 2023).

##### Preliminary conservation status.

This new species is known from one locality, situated in the unprotected limestone area, and more than 500 individuals (including many mature and young plants) were found. It may be considered as ‘Vulnerable’ (VU) under the IUCN (2001) categories and criteria D1.

##### Additional specimens examined.

China. Guangdong Province, Yangchun City, Chunwan Town, Nali village, in limestone hills, 22.410809°N, 111.932152°E, alt. 200 m., 21 March 2023, *You-Sheng Chen, Zhu-Qiu Song, Bu-Yun Zhang & Zhen Wang YC20230221* (IBSC).

## ﻿Discussion

In China, *Phyllanthodendron* was usually accepted as a distinct genus, and 10 species were recorded in the limestone area (Li 1987, 1994; Wei 2005; Li and Gilbert 2008; Xia and Tong 2018). Yao et al. (2021) synonymized *Phyllanthodendronorbicularifolium* P.T. Li under *P.petraeum* P.T. Li, because they found a continuous variation in the characters that have been used to distinguish them through examination of herbarium specimens and field observations. Ding et al. (2023) newly reported *Phyllanthusmirabilis* Müll. Arg. (= *Glochidionmirabile* (Müll. Arg.) R.W. Bouman) from China. Thus, 11 species (including *G.yangchunense*) of the previous *Phyllanthodendron* are distributed in China. Due to these changes, a new key for the 11 species in China is provided here.

Morphologically, in the previous *Phyllanthodendron*, the sepals of most species are concave inside and form a cup-shaped or urceolate shape, and the shape of disc segments is usually described as linear, oblong, or ligulate (Li and Gilbert 2008; Bouman et al. 2022; also see Fig. 3). But the new species described in this study, *Glochidionyangchunense*, is a peculiar species and it has the sepals jointly to a discoid shape, and T-shaped disc segments (Fig. 1F, H). The new species resembles *Glochidionanthopotamicum*, a species widely distributed from southwestern China to southeastern China (Li and Gilbert 2008). However, the new species differs greatly from the latter by a series of morphological characters that can be referenced from the above chapter “Diagnosis”. Additionally, the new species usually has larger leaves at the upper part of branches and smaller leaves at the lower part of branches (Fig. 1C, E), and sepals acute to slightly acuminate at apex (Fig. 1F, H). While the species *G.anthopotamicum* usually has smaller leaves at the upper part of branches and larger leaves at the lower part of branches, and sepals caudate-acuminate at apex (Fig. 3C–F). The new species is also similar to *G.dunnianus* (H. Lév.) R.W. Bouman in having evident lateral veins in sepals (especially in female flowers; Figs 1F, H, 3K, L), but it differs from the latter by its terete branches (Fig. 1C, E), outer sepals much narrower than inner ones (Fig. 1F, H), anther connectives with an apiculate apex (Fig. 1I), T-shaped disc segments (Fig. 1F, H), and smaller fruits (ca. 8–10 mm in diameter; Fig. 1K). In contrast, *G.dunnianus* has 2-winged branches (Fig. 3H, I), equivalent or sub-equivalent sepals (Fig. 3K, L), anther connectives with a narrowly subulate apex (Fig. 3L), linear-shaped disc segments (Fig. 3K, L), and larger fruits (ca. 10–15 mm in diameter).

### ﻿Key to *Glochidionyangchunense* and related species in China

**Table:** 

1	Sepals forming discoid or broadly campanulate	**2**
–	Sepals forming urceolate or cup-shaped	**4**
2	Sepals forming discoid; disc segments T-shaped; stipules ovate-triangular, ca. 1 mm long	***G.yangchunense* Z.Q. Song & Gang Yao**
–	Sepals forming broadly campanulate; disc segments linear; stipules lanceolate, 3–5 mm long	**3**
3	Branchlets prominently winged; sepals with reticular lines	***G.dunnianus* (H. Lév.) R.W. Bouman**
–	Branches terete; sepals without reticular lines	***G.petraeum* (P.T. Li) R.W. Bouman**
4	Branches angulose or prominently winged, glabrous or pubescent	**5**
–	Branches terete, pubescent	**8**
5	Branches pubescent; leaf blades oblong, oblique at base, obtuse at apex; fruiting pedicels less than 1 cm long	***G.breyniopsis* Esser & R.W. Bouman**
–	Branches glabrous; leaf blades lanceolate, symmetrical at base, acuminate to caudate at apex; fruiting pedicels 3–4 cm long	**6**
6	Ovaries and fruits glabrous	***G.caudatifolium* (P.T. Li) R.W. Bouman**
–	Ovaries and fruits pubescent	**7**
7	Male sepals and disk segments 5 or 6, stamens 3	***G.lativenium* (Croizat) R.W. Bouman**
–	Male sepals, disk segments, and stamens 4	***G.moi* (P.T. Li) R.W. Bouman**
8	Leaf base obliquely cordate; fruits triangular-globose	***G.mirabilis* (Müll. Arg.) R.W. Bouman**
–	Leaf base cuneate or rounded; fruits subglobose	**9**
9	Ovaries and fruits glabrous; fruiting pedicels less than 1 cm long	***G.anthopotamicum* (Hand.-Mazz.) R.W. Bouman**
–	Ovaries and fruits pubescent fruiting pedicels more than 3–4 cm long	**10**
10	Leaf blades 6–12 cm long	***G.yunnanense* (Croizat) R.W. Bouman**
–	Leaf blades 11.5–23.5 cm long	***G.roseum* (Craib & Hutch.) R.W. Bouman**

## Supplementary Material

XML Treatment for Glochidion yangchunense

## ﻿Additional information

### Author ORCIDs

Zhu-Qiu Song https://orcid.org/0000-0002-7763-2431

Gang Yao https://orcid.org/0000-0003-3101-8230

### Data availability

All of the data that support the findings of this study are available in the main text.
